# Standardization in laboratory medicine: Two years’ experience from category 1 EQA programs in Spain

**DOI:** 10.11613/BM.2019.010701

**Published:** 2018-12-15

**Authors:** Carmen Ricós, Carmen Perich, Beatriz Boned, Elisabet González-Lao, Jorge Diaz-Garzón, Montserrat Ventura, Sandra Bullich, Zoraida Corte, Joana Minchinela, Fernando Marques, Margarita Simón, Virtudes Alvarez, José-Vicente García-Lario, Pilar Fernández-Fernández, Pilar Fernández-Calle

**Affiliations:** 1 Spanish Society of Laboratory Medicine (SEQCML), Analytical Quality Commission, Barcelona, Spain; 2 Clinical Laboratory Department, Vall d’Hebron Barcelona Hospital Campus, Barcelona, Spain; 3 Aragonese Health Service, Royo Villanova Hospital, Zaragoza, Spain; 4 Quality Healthcare Consulting, ACMS Group, Madrid, Spain; 5 La Paz University Hospital, Madrid, Spain; 6 External Quality Assurance Programs, SEQCML, Barcelona, Spain; 7 Clinical Analysis Service, Hospital San Agustin, Aviles, Principality of Asturias, Spain; 8 Metropolitana Nord Unified Laboratory (LUMN), Germans Trias I Pujol University Hospital, Badalona, Spain; 9 Department of Clinical Biochemistry, University Hospital of Salamanca, Salamanca, Spain; 10 Intercomarcal laboratory consortiums of Alt Penedès, Anoia and Garraf, Barcelona, Spain; 11 Clinical Laboratory, Hospital Campus de la Salud, Granada, Spain

**Keywords:** standardization, external quality assessment, traceability, bias

## Abstract

**Introduction:**

Standardization is the ability to obtain interchangeable results leading to same medical interpretation. External quality assessment (EQA) is the main support of the on-going harmonization initiatives. Aim of study was to evaluate results obtained from two years category 1 EQA program experience in Spain and determine the impact of applying this type of EQA program on the analytical standardization.

**Materials and methods:**

According to the analytical method, traceability and instrument different groups were established which results were evaluated by calculating mean, coefficient of variation and percent of deviation to the reference value. Analytical performance specifications used to the results' evaluation were derived from biological variation for bias and from the inter-laboratory coefficients of variation found in a previous pilot study.

**Results:**

Only creatinine measured by enzymatic methods gave excellent results, although few laboratories used this method. Creatine kinase and GGT gave good precision and bias in all, but one instrument studied. For the remaining analytes (ALT, ALP, AST, bilirubin, calcium, chloride, glucose, magnesium, potassium, sodium, total protein and urate) some improvement is still necessary to achieve satisfactory standardization in our setting.

**Conclusions:**

The two years of category 1 EQA program experience in Spain have manifested a lack of standardization of 17 most frequent biochemistry tests used in our laboratories. The impact of the information obtained on the lack of standardization is to recommend abandoning methods such as ALT, AST without exogenous pyridoxal phosphate, Jaffe method for creatinine, and do not use non-commutable calibrators, such as aqueous solutions for calcium and sodium.

## Introduction

The main objective of clinical laboratory is to provide clear, reliable and useful information for clinical decision-making. Current healthcare systems imply performing laboratory tests in different locations, so standardization among laboratories become one of the cornerstones of the quality patient‘s care. Standardization can be defined as the ability to obtain interchangeable results (within certain analytical quality uncertainty) in order to achieve the same medical decision, regardless of the analytical procedure (method, traceability and instrument), measurement units and reference intervals.

The standardization should be based on six basic pillars, which include *in vitro* diagnostic companies, reference materials, reference methods, reference laboratories, medical laboratories and external quality assessment (EQA) organizations (*1*). Recently, Greaves noted that EQA is not just a pillar but the central support for on-going harmonization (*2*). Discordance in results between laboratories and methods should become a practice no longer accepted.

It is widely accepted that the best strategy to organize an EQA scheme is to use fresh frozen commutable control samples with values assigned by reference laboratories using reference methods, which can be found on www.harmonization.net (*3*, *4*).

Spanish Society of Laboratory Medicine (SEQCML) is a non-profit scientific organization that has been providing EQA schemes in Spain since 1980 by using stabilized control materials. Since 2013 a category 1 program has been organized for basic biochemistry analytes. According to Miller *et al.* this kind of program distributes commutable control materials with reference-measurement procedure (RMP) assigned values and replicate samples in surveys are tested (*3*). Accuracy of individual laboratories is assessed by comparison with the RMP, while reproducibility is checked both intra- and inter-laboratory, and standardization is assessed by comparison of measurement procedure calibration traceability with RMP. Two initial surveys were performed in 2013 and 2014, as preliminary experiences and regular annual surveys have been organized since 2015. For a proper assessment of bias, having adequate information of measurement’s traceability is therefore a crucial point (*5*, *6*).

Another important aspect to consider is the analytical performance specification (APS) or acceptability limits selected for the evaluation of the derived results. When APS are based on biological variation (BV), it is highly recommended to use the gradual classification of APS according to its strictness: optimal, desirable and minimal (*7*). It should be noted that the APS grade could be selected according to the limitations of the current state of the art, being defined as the performance achieved by about 80% of laboratories. According to this criterion, in this study the minimal BV-based APS grade was selected for electrolytes evaluation, while desirable BV APS were chosen for enzymes and substrates.

In this regard, a performance worse than the minimum APS should alert the laboratory that its results could be at risk and clinical decision-making might be detrimentally affected. Likewise, a performance reaching the minimal grade suggest that further improvement may be beneficial for patients (*8*, *9*).

The aim of this work is to evaluate the results obtained from two years category 1 EQA program, 2015 and 2016 surveys, performed in our country and to assess the impact of applying this kind of EQA program over the analytical standardization. Evaluation is based on the inter-laboratory imprecision and the bias of the peer group means compared with the reference method values.

## Materials and methods

Commutable control materials were purchased from MCA laboratory (Queen Beatrix Hospital, Winterswijk, The Netherlands) by means of the Stichting Kwaliteitsbewaking Medische Laboratorium Diagnostiek (SKML). According to Cobbaert *et al*. controls had been prepared from fresh anonymized left-over sera of routine laboratory with exclusion of lipemic, icteric, positive hepatitis B surface antigen (HBsAG), human immunodeficiency virus (HIV) and hepatitis C virus (HCV) samples, and stored frozen at – 84 ºC in aliquots. Pathological concentration ranges were created by adequately mixing pools and by spiking with minerals, recombinant human enzymes and human albumin (*10*). Commutability had been verified by SKML, as explained by Baadenhuijsen *et al.* and Jansen *et al.* (*11*, *12*). Throughout the years commutability has been monitored by including a native, single donation spy-sample (*10*, *12*).

Six vials of fresh frozen human serum pools at different concentrations were distributed once *per* year in a single express shipment at – 80 ºC and delivered within 24 hours to laboratories all over Spain. Different lots at different concentrations were provided for each of the two surveys. Participant laboratories were requested to maintain samples at – 20 ºC until analysis, which had to be performed within the following 14 days. Each vial had to be analysed in duplicate, one vial *per* day, for 6 consecutive days whenever possible. Results were registered on the SEQCML-EQA website, in order to be either individually and globally evaluated.

A preliminary 2013 survey was carried out in 19 laboratories and was addressed to ascertain whether the logistics of managing a non-stabilized set of control materials was operative in our country. No incidents were observed with temperature maintenance during the time between deliveries of control materials from the provider to the laboratory analysis.

Another point of interest of this preliminary survey was to explore whether laboratories could adequately inform about their analytical traceability to standards. Important difficulties were perceived that impelled holding a meeting between EQAs organization and providers, claiming for clear and complete information on calibrators’ traceability.

In 2014 first survey was performed, as part of a pilot European study (INPUTs) (Italy, The Netherlands, Portugal, Spain and The United Kingdom), with a total of 20 laboratories participants and whose results has been already published (*12*, *13*). Only about 45% of participants were able to correctly inform about its traceability, so results are not shown in this study. This survey was then considered as a pilot to identify the problems that could impact on the EQA participation and further interpretation of results. For both surveys as well as for those performed in 2015 and 2016, same sample management protocol was applied.

The 2015 and 2016 surveys were exclusively run in Spain and included 17 analytes. The number of registered participants was 93 and 105, respectively. The target values of distributed control materials were assigned by the reference methods and laboratories (Table 1).

**Table 1:** Analytes, reference methods and laboratories used to assign values

**Analytes**	**Reference method**	**Reference laboratory**
***Electrolytes***		
Calcium	Atomic Absorption Spectrometry	INSTAND eV. Düsseldorf, Germany
Chloride	ICP-IDMS	
Magnesium		
Potassium		
Sodium		
***Substrates***		
Bilirubin	Doumas method	DGKL, Hannover, Germany
Creatinine	IDMS	DGKL, Bonn, Germany
Glucose	GC-IDMS	INSTAND eV. Düsseldorf, Germany
Protein	Modified Biuret	
Urate	HPLC	Erasmus Medical Centre, Rotterdam, Netherlands
***Enzymes***		
ALP	IFCC	Unknown
α-Amylase		Haga Hospital, The Netherlands
AST		
ALT		
CK		
GGT		
LD		
The Doumas method according to Rainer *et al.* (*14*). ICP-IDMS - Inductively Coupled Plasma-Isotope Dilution Mass Spectrometry. DGKL - German Society for Clinical Chemistry and Laboratory medicine. IDMS - Isotope Dilution Mass Spectrometry. GC-IDMS - Gas Chromatography - Isotope Dilution Mass Spectrometry. HPLC - High Performance Liquid Chromatography. ALP: Alkaline phosphatase. ALT - alanine aminotransferase. AST - aspartate aminotransferase. CK - creatine kinase. GGT – gamma glutamyl transferase. LD - lactate dehydrogenase. IFCC - International Federation of Clinical Chemistry.		

Results were categorized by measurement procedure, traceability and instrument. The description of standard materials used by participants for calibration traceability is shown in Table 2. Participant laboratories using the same combination of these three elements were considered as a peer group. The peer groups and the number of laboratories included for each analyte are shown in Figures 1-17.

**Table 2:** Description of standards used by participating laboratories

**Standard**	**Traceability**
ERM-AD 452 / IFCC	Animal tissue. Non commutable
ERM-AD 455 / IFCC	Lyophilized human serum. Commutability not proven
ERM-AD 453 / IFCC	Animal tissue. Non commutable
IRMM / IFCC 456	Human tissue. Commutability not proven
NIST SRM 909 a,b	Lyophilized human serum. Commutability not proven
NIST-SRM 915	Calcium carbonate
NIST SRM 918b	Potassium chloride
NIST SRM 919b	Sodium chloride
NIST SRM 929	Magnesium gluconate
NISTSRM 956, 965	Frozen human serum. Commutability not proven
NISTSRM 967	Frozen human serum. Commutable
NIST SRM 2201	Sodium chloride in aqueous solution
NIST SRM 2202	Potassium chloride in aqueous solution
Reference materials and analytes (involved in this study) associated: ERM-AD 452 / IFCC: gamma glutamyl transferase. ERM-AD 455 / IFCC: creatine kinase. ERM-AD 453 / IFCC: lactate dehydrogenase. NIST SRM 909 a,b: calcium, chloride, creatinine, magnesium, potassium, sodium, urate. NIST SRM 915: calcium. NIST SRM 918b: potassium. NIST SRM 919b: sodium. NIST SRM 929: magnesium. NIST-SRM 956: calcium, magnesium, potassium, sodium. NIST SRM 965: glucose. NIST SRM 967: creatinine. NIST SRM 2201: sodium. NISTSRM 2202: potassium. IRMM - Institute for Reference Materials and Measurements. IFCC - International Federation of Clinical Chemistry.	

**Figure 1:**
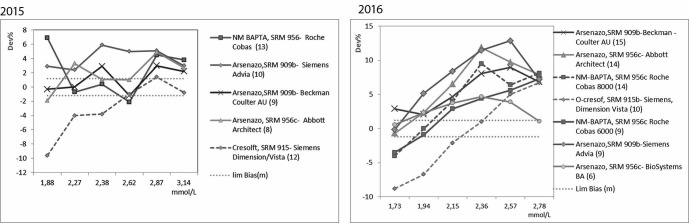
Calcium. Percentage deviation (Dev%) of peer group means from the reference value. X axis shows reference values of the six control materials. Y axis shows percent deviation of peer group mean *versus* the reference value. Numbers in brackets mean the number of participant laboratories. Lim Bias (m): acceptability limit for bias based on BV, minimum grade. NM-BAPTA: calcium specific amino-polycarboxylic acid.

**Figure 2:**
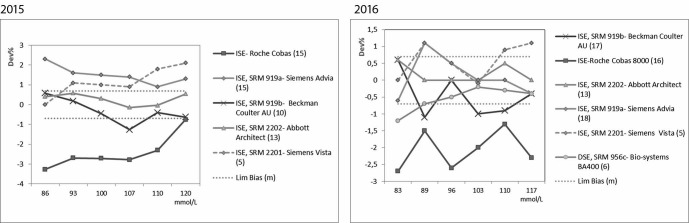
Chloride. Percentage deviation (Dev%) of peer group means from the reference value. X axis shows reference values of the six control materials. Y axis shows percent deviation of peer group mean *versus* the reference value. Lim Bias (m): acceptability limit for bias based on BV, minimum grade. ISE - ion selective electrode. Numbers in brackets indicate the laboratories participating for each instrument.

**Figure 3:**
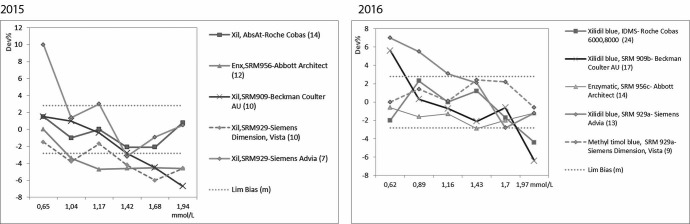
Magnesium. Percentage deviation (Dev%) of peer group mean from the reference value. X axis shows reference values of the six control materials. Y axis shows percent deviation of peer group mean *versus* the reference value . Lim Bias (m): acceptability limit for bias based on BV, minimum grade. Xil - Xilidil blue. Numbers in brackets indicate the laboratories participating for each instrument.

**Figure 4:**
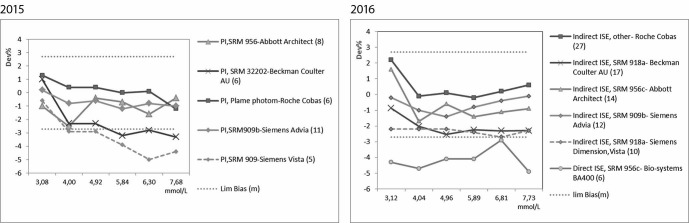
Potassium. Percentage deviation (Dev%) of peer group mean from the reference value. X axis shows reference values of the six control materials. Y axis shows percent deviation of peer group mean *versus* the reference value. Lim Bias (m): acceptability limit for bias based on BV, minimum grade. ISE - ion selective electrode. Numbers in brackets indicate the laboratories participating for each instrument.

**Figure 5:**
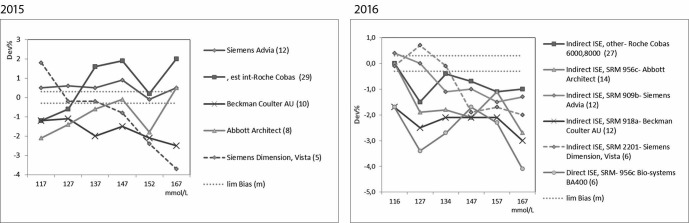
Sodium. Percentage deviation (Dev%) of peer group mean from the reference value. X axis shows reference values of the six control materials. Y axis shows percent deviation of peer group mean *versus* the reference value. Lim Bias (m): acceptability limit for bias based on BV, minimum grade. ISE - ion selective electrode. Numbers in brackets indicate the laboratories participating for each instrument.

**Figure 6:**
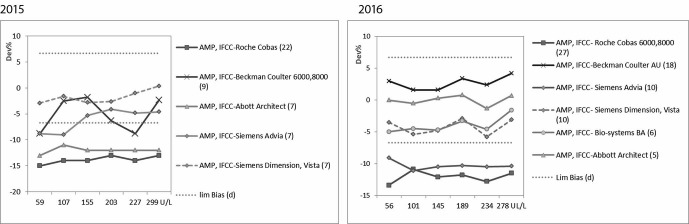
Alkaline phosphatase. Percentage deviation (Dev%) of peer group means from the reference value. X axis shows reference values of the six control materials. Y axis shows percent deviation of peer group mean *versus* the reference value. Lim Bias (d): acceptability limit for bias based on BV, desirable grade. AMP - 2-amino-2-methyl-1-propanol. Numbers in brackets indicate the laboratories participating for each instrument.

**Figure 7:**
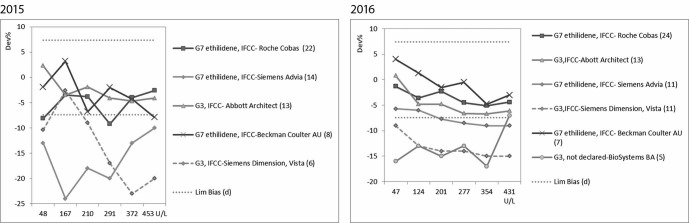
Amylase. Percentage deviation (Dev%) of peer group mean from the reference value. X axis shows reference values of the six control materials. Y axis shows percent deviation of peer group mean *versus* the reference value. Lim Bias (d): acceptability limit for bias based on BV, desirable grade. G3 - malto trioside. G7 - malto-heptaoside. Numbers in brackets indicate the laboratories participating for each instrument.

**Figure 8:**
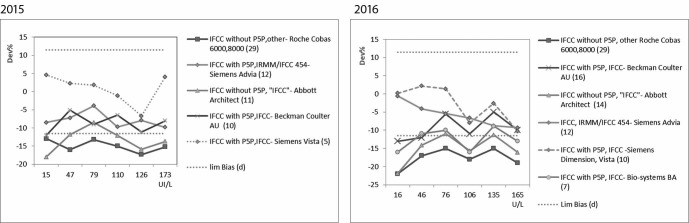
ALT. Percentage deviation (Dev%) of peer group means from the reference value. X axis shows reference values of the six control materials. Y axis shows percent deviation of peer group mean *versus* the reference value. Lim Bias (d): acceptability limit for bias based on BV, desirable grade. Numbers in brackets indicate the laboratories participating for each instrument.

**Figure 9:**
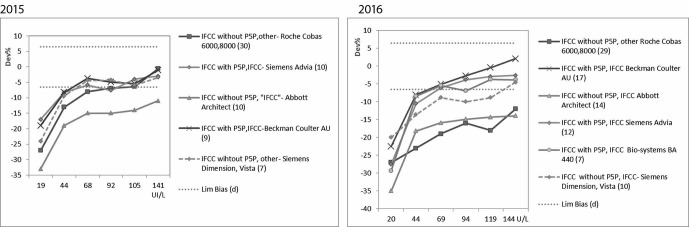
AST. Percentage deviation (Dev%) of peer group means from the reference value. X axis shows reference values of the six control materials. Y axis shows percent deviation of peer group mean *versus* the reference value. Lim Bias (d): acceptability limit for bias based on BV, desirable grade. P5P -pyridoxal-5-phosphate. Numbers in brackets indicate the laboratories participating for each instrument.

**Figure 10:**
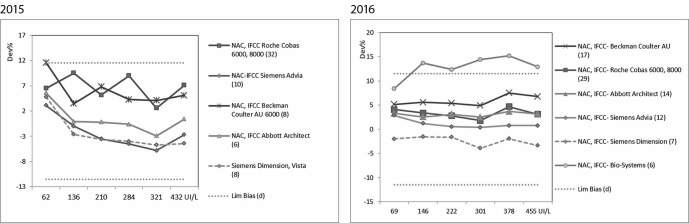
Creatine kinase. Percentage deviation (Dev%) of peer group mean from the reference value. X axis shows reference values of the six control materials. Y axis shows percent deviation of peer group mean *versus* the reference value. Lim Bias (d): acceptability limit for bias based on BV, desirable grade. NAC - N-acetyl-cysteine. Numbers in brackets indicate the laboratories participating for each instrument.

**Figure 11:**
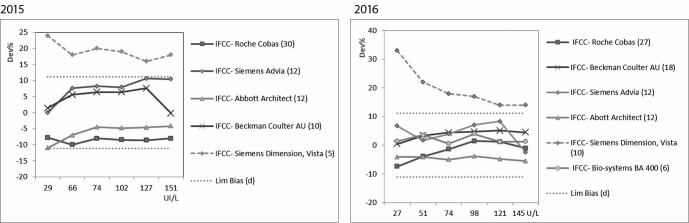
Gamma glutamyl transferase. Percentage deviation (Dev%) of peer group mean from the reference value. X axis shows reference values of the six control materials. Y axis shows percent deviation of peer group mean *versus* the reference value. Lim Bias (d): acceptability limit for bias based on BV, desirable grade. All groups use substrate: γ glutamyl-3carboxy-4nitroanilide > 4mmol/L. The exception is: Siemens Dimension, Vista that uses substrate < 4mmol/L. Numbers in brackets indicate the laboratories participating for each instrument.

**Figure 12:**
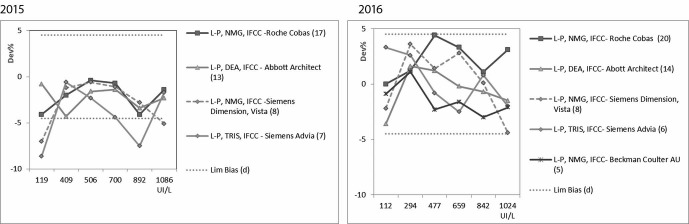
Lactate dehydrogenase. Percentage deviation (Dev%) of peer group mean from the reference value. X axis shows reference values of the six control materials. Y axis shows percent deviation of peer group mean *versus* the reference value. Lim Bias (d): acceptability limit for bias based on BV, desirable grade. NMG - N-methyl-D-glucamine. DEA - diethanolamine. TRIS -hydroxymethyl-aminomethane. Numbers in brackets indicate the laboratories participating for each instrument.

**Figure 13:**
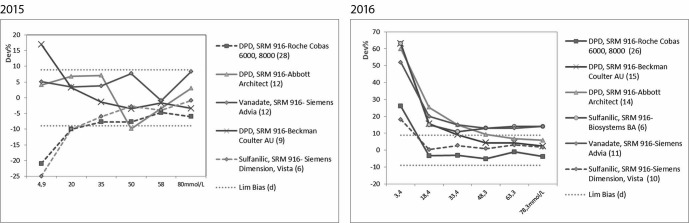
Bilirubin. Percentage deviation (Dev%) of peer group mean from the reference value. X axis shows reference values of the six control materials. Y axis shows percent deviation of peer group mean *versus* the reference value. Lim Bias (d): acceptability limit for bias based on BV, desirable grade. DPD - 3,5-dicholorophenyl-diazonium- tetrafluoroborate. Numbers in brackets indicate the laboratories participating for each instrument.

**Figure 14:**
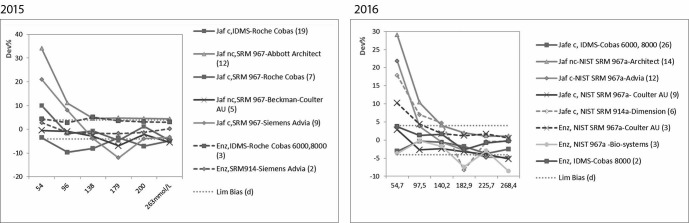
Creatinine. Percentage deviation (Dev%) of peer group mean from the reference value. Methods in figure appearing according the following order: enzymatic, compensated and non-compensated. X axis shows reference values of the six control materials. Y axis shows percent deviation of peer group mean *versus* the reference value. Lim Bias (d): acceptability limit for bias based on BV, desirable grade. Numbers in brackets indicate the laboratories participating for each instrument.

**Figure 15:**
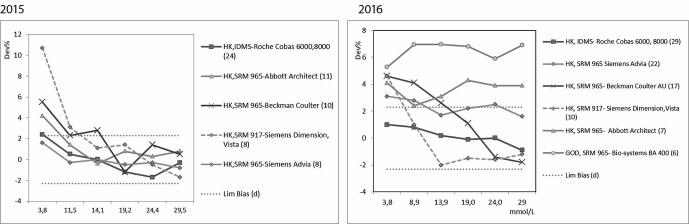
Glucose. Percentage deviation (Dev%) of peer group mean from the reference value. X axis shows reference values of the six control materials. Y axis shows percent deviation of peer group mean *versus* the reference value. Lim Bias (d): acceptability limit for bias based on BV, desirable grade. GOD - glucose oxidase. HK - hexokinase. Numbers in brackets indicate the laboratories participating for each instrument.

**Figure 16:**
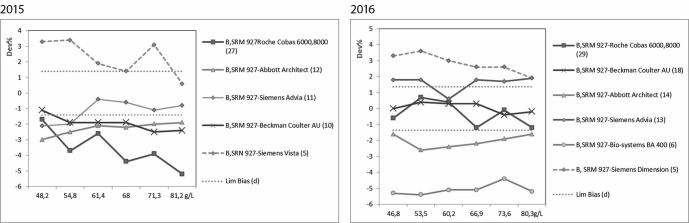
Total protein. Percentage deviation (Dev%) of peer group mean from the reference value. X axis shows reference values of the six control materials. Y axis shows percent deviation of peer group mean *versus* the reference value. Lim Bias (d): acceptability limit for bias based on BV, desirable grade. B - biuret. Numbers in brackets indicate the laboratories participating for each instrument.

**Figure 17:**
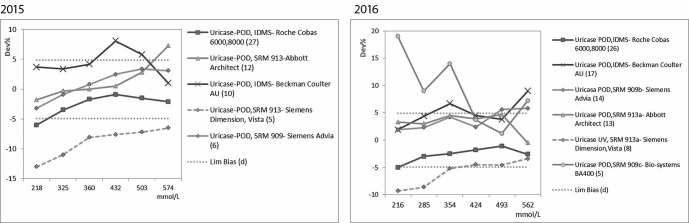
Urate. Percentage deviation (Dev%) of peer group mean from the reference value. X axis shows reference values of the six control materials. Y axis shows percent deviation of peer group mean *versus* the reference value. Lim Bias (d): acceptability limit for bias based on BV, desirable grade. POD – peroxidase. Numbers in brackets indicate the laboratories participating for each instrument.

Compared to 2015, a new instrument was incorporated in 2016 survey (Bio-systems BA 400), with only 6 participating laboratories. The overall evaluation of the 2015 survey was published on the SEQCML website and was presented at the 2016 EQALM annual meeting (*13*, *15*). Only groups formed by 5 or more final laboratories were considered in this study.

Inter-laboratory imprecision was calculated by averaging the coefficient of variation (CV) obtained from the six controls distributed on the 2016 and 2015 surveys and compared with the best (Dutch) inter-laboratory CV derived from the 2014 pilot study, which used similar six commutable control materials (*16*).

Bias was calculated by the percent difference between the peer group mean (same measurement procedure, traceability and instrument) and the reference value. The analytical performance specification to apply for bias evaluation was based on the BV data collected on the online 2014 database, which had been elaborated as detailed by Ricós *et al.*, applying the minimum level of requirement for electrolytes and the desirable level for substrates and enzymes (*17*-*19*).

The results of this study were examined with the particular focus on the most common analytical procedures used in Spain and its repercussion on non-comparable results, detected throughout participation on level 1 EQA schemes.

Standardization is defined by the attainment of inter-laboratory imprecision within the predefined APS and peer group bias (% mean deviation to the reference value) below the allowed bias derived from BV.

## Results

All results exceeding the mean ± 3 standard deviation of each group were rejected as outliers. The number of rejected participant laboratories was 5 for the 2015 survey and 10 for the 2016 survey. Moreover, 30 results for lactate dehydrogenase (LD) which were 100% higher than the others due to the different substrate (pyruvate instead of lactate) were also excluded from the study. Results for bias are presented in Figures 1-17. Results for the inter-laboratory imprecision of each peer group for electrolytes, enzymes and substrates are presented in Tables 3-5 and compared with the APS for inter-laboratory imprecision (APSIL) from the pilot 2014 survey (*16*). An overview of the standardization achieved in our setting, according to the bias and the imprecision calculated for instruments, is presented in Table 6.

**Table 3:** Inter-laboratory imprecision for electrolytes

**Calcium**	**2015, CV (%)**	**2016, CV (%)**	**APS_IL_**
Arsenazo, SRM 909b - Beckman Coulter AU	1.2	4.4	3.5
Arsenazo, SRM 909b - Siemens Advia	1.6	6.2	
Arsenazo, SRM 915a - Abbott Architect	1.2	/	
Arsenazo, SRM 956c - Abbott Architect	2.4	5.9*	
NM BAPTA, SRM 956 - Roche Cobas	1.7	1.6	
Cresolftalein, SRM 915 – Siemens Dimension, Vista	2.7	2.4	
Arsenazo, SRM 956c – Bio-systems BA	/	2.6	
**Chloride**			
ISE, SRM2202 – Abbott Architect	0.6	1.4	1.4
ISE, Gravimetry– Roche Cobas	2.1	1.5	
ISE, SRM 919 – Siemens Advia	0.7	0.6	
ISE, SRM 2201 – Siemens Vista	0.6	2.3*	
ISE, SRM 919 - Beckman Coulter AU	1.4	0.7	
DSE, SRM 956c-Bio-systems BA400	/	0.6	
**Magnesium**			
Xilidil blue, SRM 929 - Siemens Advia	11.7*	1.0	4.5
Enzymatic, SRM 956 - Abbott Architect	4.1	2.1	
Xilidil blue, SRM 909 - Beckman Coulter AU	5.3	2.5	
Xilidil blue, Atomic Absortion -Roche Cobas	8.5	3.0	
Xilidil blue, SRM929 - Siemens Dimension, Vista	6.2	2.2	
**Potassium**			
ISE, SRM 956-Abbott Architect	0.9	1.2	1.5
ISE, SRM 2202-Beckman Coulter AU	0,6	0.8	
ISE, Gravimetry-Roche Cobas	0.9	1.0	
ISE, SRM909b-Siemens Advia	0.7	0.3	
ISE, SRM 909-Siemens Vista	0.8	0,3	
DSE, SRM 956c-Bio-systems BA400	/	1.7	
**Sodium**			
ISE,SRM 956-Abbott Architect	0.4	0.9	2.1
ISE, SRM 32202-Beckman Coulter AU	0.6	0.7	
ISE,Gravimetry-Roche Cobas	1.7	0.9	
ISE, SRM 909b - Siemens Advia	0.5	0.3	
ISE,SRM 909-Siemens Vista	0.5	0.6	
DSE, SRM 956c-Bio--systems BA400	/	0.4	
*exceeding APS_IL_. The coefficient of variation (CV) is presented as the group’s average for six controls. DSE - direct selective electrode. ISE - indirect selective electrode. APS_IL_ -analytical performance specifications for inter-laboratory imprecision.			

**Table 4:** Inter-laboratory imprecision for enzymes

**ALP**	**2015, CV (%)**		**2016, CV (%)**	**APS_IL_**
4PNP-AMP, IFCC-Abbott Architect	1.9		/	6.4
4PNP-AMP, IFCC-Beckman Coulter AU	2.5		5.8	
4PNP-AMP, IFCC-Roche Cobas	1.2		3.7	
4PNP-AMP, IFCC-Siemens Dimension,Vista	3.3		4.2	
4PNP-AMP, IFCC-Siemens Advia	3.0		5.5	
AMP, IFCC- Bio-systems BA	/		11.7*	
**ALT**				
IFCC with P5P, IRMM/IFCC 454- Siemens Advia		14.1*	15.7*	8.7
IFCC without P5P, “IFCC”- Abbott Architect		14.3*	6.7	
IFCC with P5P, IFCC- Beckman Coulter AU		13.2*	9.5	
IFCC without P5P, other- Roche Cobas 6000,8000		15.0*	3.4	
IFCC with P5P, IFCC- Siemens Vista		17.0*	8.1	
IFCC with P5P- IFCC Bio-systems BA		/	10.4	
**Amylase**				
G3, IFCC- Abbott Architect		2.5	6.3	12.0
G7 ethilidene, IFCC- Roche Cobas		3.7	5.6	
G7 ethilidene, IFCC-Siemens Advia		9.7	0.6	
G7 ethilidene, IFCC-Beckman Coulter AU		2.5	3.2	
G3, IFCC-Siemens Dimension, Vista		6.2	4.6	
G3, not declared-Bio-systems BA		/	8.8	
**AST**				
IFCC with P5P, IRMM/IFCC 454- Siemens Advia		6.4	4.2	6.0
IFCC without P5P, “IFCC”- Abbott Architect		3.0	3.2	
IFCC with P5P, IFCC- Beckman Coulter AU		1.4	2.1	
IFCC without P5P,other- Roche Cobas 6000,8000		4.5	8.7	
IFCC with P5P, IFCC- Siemens Vista		6.0	5.6	
IFCC with P5P, IFCC - Bio-systems BA		/	4.0	
**CK**				
NAC, IFCC - Abbott Architect		2.2	3.7	4.9
NAC, IFCC – Beckman Coulter AU		3.9	2.6	
NAC, IFCC - Roche Cobas 6000,8000		7.4	4.5	
NAC, IFCC - Siemens Advia		2.6	2.8	
NAC, IFCC - Siemens Dimension, Vista		3.7	2.7	
NAC, IFCC - Bio-systems		/	2.6	
**GGT**				
IFCC- Abbott Architect		1.1	4.2	12.0
IFCC- Beckman Coulter AU		1.2	2.2	
IFCC- Roche Cobas		3.6	2.2	
IFCC- Siemens Advia		10.0	4.3	
IFCC- Siemens Dimension, Vista		2.9	1.3	
IFCC- Bio-systems BA 400		/	6.5	
**LD**				
L-P, DEA, IFCC - Abbott Architect		2.6	6.0	6.1
L-P, NMG, IFCC - BeckmanCoulter AU		/	10.0*	
L-P, NMG, IFCC -Roche Cobas		2.4	9.4*	
L-P, TRIS, IFCC - Siemens Advia		5.7	7.3*	
L-P, NMG, IFCC -Siemens Dimension, Vista		3.6	7.6*	
*exceeding APS_IL_. The coefficient of variation (CV) is presented as the group’s average for six controls. ALP - Alkaline phosphatase. ALT - alanine aminotransferase. AST - aspartate aminotransferase. CK - creatine kinase. GGT – gamma glutamyl transferase (substrate > 4 mmol/L only). LD - lactate dehydrogenase (substrate lactate to pyruvate only). APS_IL_ - analytical performance specifications for inter-laboratory imprecision. 4PNP – 4-p-nitrophenyl phosphate. AMP - 2-amino-2-methyl-1-propanol. P5P -pyridoxal-5-phosphate. IRMM - Institute for Reference Materials and Measurements. NAC - N-acetyl-cisteine. L-P - lactate to pyruvate. DEA – diethanolamine. NMG - N-methyl-D-glucamine. TRIS - hydroxymethyl-aminomethane.				

**Table 5:** Inter-laboratory imprecision for substrates

**Bilirubin**	**2015, CV (%)**	**2016, CV (%)**	**APS_IL_**
DPD, SRM 916-Abbott Architect	3.8	4.7	9.6
DPD, SRM 916-Beckman Coulter AU	2.3	4.8	
DPD, SRM 916-Roche Cobas 6000, 8000	1.8	15.7*	
Vanadate, SRM 916- Siemens Advia	5.1	1.1	
Sulfanilic, SRM 916- Siemens Dimension, Vista	5.3	2.5	
Sulfanilic, SRM 916-Biosystems BA	/	6.5	
**Creatinine**			
Jaf nc, SRM 967-Abbott Architect	1.4	2.0	7.0
Jaf nc, SRM 967-Beckman-Coulter AU	7.7	5.8	
Jaf c, IDMS – Roche Cobas6000, 8000	2.4	3.6	
Jaf c, SRM 967-Roche Cobas 6000, 8000	4.0	/	
Jaf c, SRM 967-Siemens Advia	3.0	1.2	
Jaf c, NIST SRM 914a – Dimension	/	1.4	
Enz, NIST SRM 967ª–Coulter AU	/	2,9	
Enz, NIST 967a –Bio-systems	/	4,0	
Enz, IDMS-Cobas 8000	/	3,1	
**Glucose**			
HK, SRM 965-Abbott Architect	5.4	4.5	5.9
HK, SRM 965-Beckman Coulter AU	2.4	3.4	
HK, IDMS-Roche Cobas 6000,8000	8.1*	0.8	
HK, SRM 965-Siemens Advia	3.8	2.5	
HK, SRM 917-Siemens Dimension, Vista	7.2*	2.0	
GOD, SRM 965- Bio-systems BA 400 (*6*)	/	2.0	
**Total protein**			
B, SRM 927 –Abbott Architect	3.2	3.2	3.2
B, SRM 927-Beckman Coulter AU	4.9	2.3	
B, SRM 927Roche Cobas 6000,8000	4.6	6.4*	
B, SRM 927-Siemens Advia	8.8*	2.0	
B, SRM 927-Siemens Vista	4.2	1.6	
B, SRM 927 - Bio-systems BA 400	/	2.0	
**Urate**			
Uricase-POD, SRM 913-Abbott Architect	3.0	3.1	5.2
Uricase-POD, IDMS- Beckman Coulter AU	3.5	3.2	
Uricase-POD, IDMS - Roche Cobas 6000,8000	3.5	1.2	
Uricase-POD, SRM 909 - Siemens Advia	2.2	2.0	
Uricase-POD, SRM 913 - Siemens Dimension, Vista	1.1	4.1	
Uricase-POD, SRM 909c - Bio-systems BA400	/	3.5	
*exceeding APS_IL_.The coefficient of variation (CV) is presented as the group’s average for six controls. Only instruments with more than 5 participating laboratories are shown in this table. APS_IL_ - analytical performance specifications for inter-laboratory imprecision. B – Biuret. DPD - 3,5-dicholorophenyl-diazoniumtetrafluoroborate. Enz – enzymatic. Jaf – Jaffe. Jaf c - Jaffe compensated. Jaf nc - Jaffe non compensated. HK – hexokinase. POD – peroxidase.			

**Table 6:** Overview of achieved results toward standardization in our setting

**Analytes**	**Architect**	**AU**	BA400*	**Cobas** **6000 and 8000**	**Advia**	**Dimension** **Vista**
ALP	TI	OK	TI	TI	TI	OK
ALT	TI	TI	TI	TI	TI	OK
Amylase	OK	OK	TI	OK	TI	TI
AST	TI	TI	TI	TI	TI	TI
Bilirubin	TI	TI	TI	TI	TI	TI
Calcium	TI	TI	TI	TI	TI	TI
Chloride	OK	TI	TI	TI	TI	OK
CK	OK	OK	TI	OK	OK	OK
Creatinine, enzymatic	-	-	-	OK	-	OK
Creatinine, Jaffe	TI	TI	TI	TI	TI	TI
GGT	OK	OK	OK	OK	OK	TI
Glucose	TI	TI	TI	TI	TI	TI
LD	OK	TI	-	TI	TI	TI
Magnesium	TI	TI	TI	TI	TI	TI
Potassium	OK	OK	TI	OK	OK	OK
Total protein	TI	TI	TI	TI	TI	TI
Sodium	TI	TI	TI	TI	TI	TI
Urate	OK	TI	TI	OK	OK	TI
TI: To improve because either bias or inter-laboratory imprecision does not reach the APS in both or in one of the two surveys evaluated. *BA400 group (Bio-systems) began its participation in the 2016 survey. Only instruments with more than 5 participating laboratories are shown in this table. ALP - alkaline phosphatase. ALT - alanine aminotransferase. AST - aspartate aminotransferase. CK - creatine kinase. GGT – gamma glutamyl transferase. LD - lactate dehydrogenase. OK: Bias and inter-laboratory imprecision achieve the APS.						

## Discussion

The percentage of laboratories excluded was higher in 2016 than in 2015 due to better knowledge of the traceability-instrument, so groups were more specific in 2016. This cannot be considered a disadvantage. The results in this study are discussed form the light of their impact on the aims proposed. These are: positive, negative and needed to be dialogued with providers.

Main positive impacts, which imply an adequate standardization not needing for further improvements, apply to potassium and creatine kinase (CK). Potassium shows inter-laboratory imprecision and bias (Figure 4) within the allowable limits for almost all peer groups. For the remaining electrolytes good inter-laboratory imprecision can also be seen, well in agreement with the 2014 survey (performed in collaboration with other European countries) where all participant laboratories and manufacturers fulfilled the APS for total analytical error at the minimum performance level (*20*). Creatine kinase show good inter-laboratory imprecision and bias (Figure 10), except for the new group enrolled in the 2016 survey (BA400). So it may be expected a well standardized measurements soon. Negative impacts may be due to several reasons. The aqueous matrix of SRM 915 and 918 used for calcium and sodium, respectively (Figures 1 and 5), produces low results. Lack of commutability of calibration traceability materials was described to be a crucial factor to assure standardization in medical laboratories by Panteghini and Ambruster (*21*, *22*).

Instrument dependent problems can be seen in this study for alkaline phosphatase (ALP) with low results for Roche users (Figure 6), whereas all participants use same method and traceability; this event causes an important lack of standardization in our country because it is the greatest group. Same results had been seen by Braga *et al*., and Aloisio *et al.* who observed discrepancies among Abbott Architect users related to an “experimental” calibration factor provided by the manufacturer (*23*, *24*). Non-standardized ALP results could have a great impact in some clinical scenarios such as hypophosphatemia diagnosis, so an improvement in the results’ traceability becomes a crucial objective (*25*). Method dependent troubles are seen in four cases.

Firstly, amylase, were all groups using malto-heptaoside (G7) substrate, as well as the malto-trioside (G3) of Abbott Architect show harmonized results. The remaining G3 groups have unacceptable negative bias (Figure 7). This lack of standardization affects one third of the participants of this study, thus producing a considerable impact on the healthcare in our country. Alanine aminotransferase (ALT) and aspartate aminotransferase (AST) testing show unacceptable inter-laboratory imprecision and bias (low results) (Figures 8 and 9) for laboratories that did not add pyridoxal-5-phosphate (P5P) in its measurement procedure. Infusino *et al.* and Jansen *et al.* reported that when reagent is supplemented with P5P the ratio of preformed holoenzyme to apoenzyme differs among specimens (*12*, *26*). Gamma glutamyl transferase (GGT), were all groups using substrate of γ-glutamyl-3carboxy-4nitroanilide > 4mmol/L have good precision and bias; however, the Siemens Dimension Vista group that uses a different concentration of substrate (< 4 mmol/L) produces unacceptable high results (Figure 11). Lastly, creatinine shows good inter-laboratory CV. However, only enzymatic methods have good bias at the entire concentration range studied, whereas most of the Jaffe based measurements produce unacceptable high results at low-normal concentrations (≤ 50 mmol/L) and some of them show inconsistent bias along the two surveys evaluated (Figure 14). Part of the 2015 results had been previously published and is in accordance with the 2016 survey, as well as with Jassam *et al.* that observed as Abbott compensated and Jaffe methods were most affected by glucose interferences, resulting in either under- or over- estimation of GFR and may also lead to errors in the classification of chronically kidney disease (*20*, *27*, *28*). Likewise, data reported by Panteghini showed an 18 μmol/L positive bias derived from the Jaffe-based method on a Beckman AU 2710 instrument (*29*). These results are especially relevant for paediatric population. Our results evidences that for consecutive years the Jaffe method produces false high results at low-normal concentration values, in all the instruments used in our country. Consequently, creatinine is not standardized in our setting and considering the clinical implications associated, Jaffe method should be abandoned. Dialogue with providers is of upmost necessity in several cases. The main negative issue is the lack of adequate information about the calibration traceability of the measurement procedure; this circumstance was observed to affect the 55% of participating laboratories in 2015. In order to address and minimize this issue, the SEQCML- Analytical Quality Commission promoted regular and specific meetings with providers and holding educational communications and workshops in national laboratory congresses (*5*, *6*). This effort seems to have been worthy, observing a decrease in the percentage of wrong-coding traceability from 55% to 20% in 2016.

Some *in vitro* diagnostic medical device providers reported their methods for ALT and AST as “IFCC traceable” when no P5P was added; this created a high incidence of wrong codifications by laboratory workers that was solved and recorded by SEQCML after informing of this circumstance to providers and users.

Lactate dehydrogenase measurements gave good inter-laboratory CV in the 2015 survey but not in 2016; the reason for this remains unknown and should be discussed with providers. Bias showed an interesting improvement, resulting in satisfactory results for all users of the lactate to pyruvate based measurement in the 2016 survey (Figure 12).

Our findings for bilirubin, chloride, glucose, magnesium (irregular inter-laboratory CV and bias), as well as total protein and urate (good inter-laboratory imprecision, but irregular bias) led us to the opinion that a dialogue with providers would be necessary for improving standardization in our country.

A limitation of this study would be the reduced number of participants in certain groups, due to the fact that this program is still poorly known by many Spanish laboratories. Consequently, one symposium, various workshops in the national congress and specific meetings were organized in 2017, a book has been written in 2018 and other educational activities are planned for the future to overcome this limitation.

Another drawback might be that there is a single exercise *per* year; this could be not enough to guarantee the trueness for the rest of the year. Because the economic difficulty to make more distributions of these controls materials along the year, laboratories in Spain could use our regular EQA schemes (stabilized materials, peer group evaluation, one sample *per* month) to verify if their analytical performance is maintained along the year.

## Conclusions

The two years of category 1 EQA program experience in our country have manifested a lack of standardization of the 17 more frequent general biochemistry tests used in our laboratories. The application of this kind of EQA program allows estimating measurement procedure-traceability-instrument bias in a way that can be expanded to what happens with real patient samples. The impact of the information obtained by category 1 EQA program on the lack of standardization is: to recommend abandoning methods such as for ALT, AST without exogenous pyridoxal phosphate, Jaffe method for creatinine, pyruvate-lactate for LD, and do not use non-commutable calibrators, such as aqueous solutions for calcium and sodium.
